# Age, sex, adult and larval diet shape starvation resistance in the Mediterranean fruit fly: an ecological and gerontological perspective

**DOI:** 10.1038/s41598-019-47010-0

**Published:** 2019-07-24

**Authors:** Christos D. Gerofotis, Nikos A. Kouloussis, Christiana Koukougiannidou, Nikos T. Papadopoulos, Petros Damos, Dimitris S. Koveos, James R. Carey

**Affiliations:** 1 Laboratory of Applied Zoology and Parasitology, School of Agriculture, Aristotle University of Thessaloniki, 54124 Thessaloniki, Greece; 2 Laboratory of Entomology and Agricultural Zoology, Department of Agriculture Crop Production and Rural Environment, University of Thessaly, Phytokou St. 38446 N, Ionia Volos, Greece; 3 Department of Entomology, University of California, Davis, CA 95616, United States; 4 Center for the Economics and Demography of Aging, University of California, CA 94720 Berkeley, United States

**Keywords:** Entomology, Ecophysiology

## Abstract

The ability of an animal to withstand periods of food deprivation is a key driver of invasion success (biodiversity), adaptation to new conditions, and a crucial determinant of senescence in populations. Starvation resistance (SR) is a highly plastic trait and varies in relation to environmental and genetic variables. However, beyond *Drosophila*, SR has been studied poorly. Exploiting an interesting model species in invasion and ageing studies-the Mediterranean fruit fly (*Ceratitis capitata*)- we investigated how age, food and gender, shape SR in this species. We measured SR in adults feeding in rich and poor dietary conditions, which had been reared either on natural hosts or artificial larval diet, for every single day across their lifespan. We defined which factor is the most significant determinant of SR and we explored potential links between SR and ageing. We found that SR declines with age, and that age-specific patterns are shaped in relation to adult and larval diet. Females exhibited higher SR than males. Age and adult diet were the most significant determinants of SR, followed by gender and the larval diet. Starvation resistance proved to be a weak predictor of functional ageing. Possible underlying mechanisms, ecological and gerontological significance and potential applied benefits are discussed.

## Introduction

All life processes and physiological events expend energy to function. A continuous supply of energy is necessary for an animal to survive and reproduce^1^. This central dogma is ubiquitous across the animal kingdom and an understanding of starvation resistance remains a central theme in modern animal biology and ecology. Starvation resistance (SR hereafter) is the ability of an animal to withstand periods of food deprivation or limitation^2^. In nature, environmental conditions are highly unpredictable as food availability varies in time and space, and virtually each animal will suffer a period of nutritional stress (that could ultimately lead to starvation-induced mortality) during its lifetime^2^. As a result, SR is an extreme form of adaptive physiology, that can force animals to produce new phenotypes with special characters and/or strategies that can mitigate the consequences of food stress^3,4^.

Stress resistance characters have been associated in many studies with fitness related traits (e.g. longevity, fecundity, body mass), aging *per se* (senescence) and colonization success and thus have great ecological and evolutionary significance. For example, selection for SR increased longevity and reduced early fecundity in *Drosophila*, while long-lived mutants/selected lines tend to be more food resistant (reviewed in^3,^^5^). Additionally, high SR has been correlated with large adult body mass^6^, and clinal variation in *Drosophila* populations has been related to SR with higher levels occurring at lower latitudes^5,7^. However, there are several studies demonstrating that SR and the above traits are not strictly positively correlated (detailed in^3^).

A large portion of these studies focuses on the association between stress resistance and longevity, investigating declines in age-related fitness traits (e.g. starvation and desiccation resistance, thermal tolerance), ultimately aiming to elucidate senescence ageing, since both of these traits seem to share similar genetic regulatory mechanisms (mentioned in^3,8^). Stress response throughout an organism’s lifespan is a critical component of fitness and modulator of lifespan^9–11^. Due to the above and apart from their ecological significance, stress factors and especially SR have attracted the interest of researchers in gerontology science.

Species and individuals differ in their ability to tolerate food stress. Within the same species, differences in SR among individuals/populations may arise from heritable genetic variation and/or as a result of a phenotypic plasticity evoked by environmental variables. Such factors (genetic and environmental) operate at the same time to shape an animal’s SR. Some of these variables have been studied extensively (due to their significance) while others have been neglected (due to difficulties). For example, Lee and co-workers in a seminal paper have elucidated the nutritional basis of SR in *Drosophila melanogaster*^12^. At the intra-population level, gender also seems to be one of the most relevant determinants of SR, as shown in *Coleoptera*^13^ and in *Drosophila* fruit flies^14^. In general, females are expected to withstand more food limitation than males and this is usually correlated to their larger body size and to differential energy demands, acquisition and utilization of nutritional resources between the two sexes (as discussed in^15^). However, this pattern is not always the same and researchers conclude that differences in SR between the two sexes vary considerably in relation to factors such as nutrition^12^, social interactions (e.g. mating)^16^, strain^17^ and age^18,19^.

In contrast, the effect of age on SR has not been explored extensively. With the exception of a paper by Shahrestani^18^ that investigates age-specific SR in *Drosophila*, most studies usually focus on comparing SR among groups of individuals (e.g. young versus older). Generally, a reduction of stress resistance with age (including starvation) is expected as a result of functional senescence^10,20^. Yet, in nature all these variables and many more, may operate simultaneously and at any given time and thus age-dependent alteration of SR can occur at any time across an animal’s adult life. To make realistic predictions on how SR is shaped and to identify the most significant factors we need studies that consider multiple variables across an animal’s lifespan.

To date, such extensive studies are limited. Insects are the main study organism for investigating SR, because they are easy to rear and maintain in the lab and most importantly no ethical or legal concerns apply to insects. Traditionally, the majority of information on SR comes from the fruit fly *Drosophila melanogaster*. Although *Drosophila* offers many advantages (reviewed in^3,21^), investigating SR in other organisms is recommended and can usefully complement the existing information, given the diversity of responses observed across the animal kingdom. The Mediterranean fruit fly (also referred as medfly) offers a unique opportunity for studying starvation. Specifically, this species is a significant invasive species worldwide and has been used extensively in ageing studies^22–24^. Therefore, a thorough research on medfly SR may provide new insights in the ecology of this species (e.g. invasion ability), while potential extrapolation of the results may contribute to a better understanding of senescence.

In this study, three questions were addressed. Firstly, how SR is shaped across the medfly’s lifespan. Secondly, whether age, sex, larval and adult diet are significant predictors of SR and which of these factors shapes starvation resistance more forcefully. Finally, whether SR can be used as a (bio) marker of ageing to identify ageing processes. Accordingly, our predictions were that SR will decline with age due to functional senescence, but the magnitude of the decrease will vary in relation to the other factors. We predict that larval diet, will be the strongest determinant of SR, followed by adult diet and gender (without considering age). Although, there is no clear evidence about which of the tested variables should be expected to impact SR more (on *Ceratitis capitata)*, we have chosen larval diet as the prevailing variable, because of the general literature on strong ontogenetic carry over effects of developmental environment to adult physiology responses. To answer the first two questions, we measured SR in medflies of both sexes across the whole adult lifespan and at the same time, we manipulated important variables such as adult diet and larval diet to investigate their impact on SR. To stress the final question, we estimated the critical time points above which abrupt (i.e. not following the general trend) changes/fluctuations in SR take place.

## Results

### Age-specific starvation resistance

**Figure 1:**
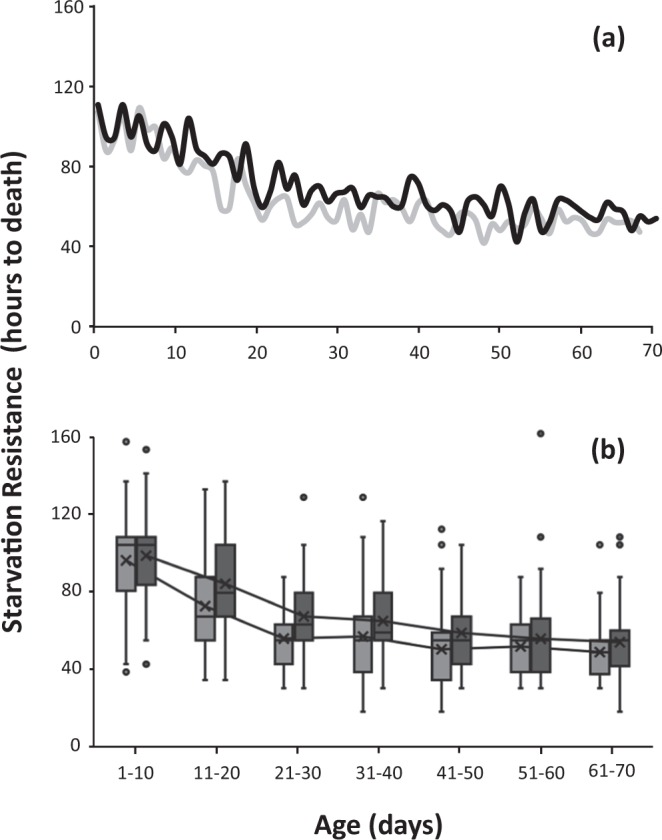
Age-specific curves (**a**) and boxplot distribution (**b**) of SR in citrus reared *Ceratitis capitata* flies kept in adult full diet. Light grey bars indicate males and dark grey indicate females respectively. Lines along the boxplots connect the mean time to death among each age class.

SR varied in relation to age for both males and females (Fig. 1) developed in citrus fruits. SR decreased with age for both sexes in medflies kept in full adult diet (Fig. 1a). A significant decrease from 110 to 60 hours in SR was observed from 1 to 35 days of age. Above 35 days SR is stabilized. This decreasing trend as flies age is also illustrated by the mean time to death line (Fig. 1b). SR differed among most of the age-classes (Wald X^2^ = 183.35, df = 6, P = 0.0001, Supplementary Table 1) and females were more resistant to food absence than males in all age-classes (X^2^ = 4.407, df = 1, P = 0.036, Supplementary Table 1).

**Figure 2:**
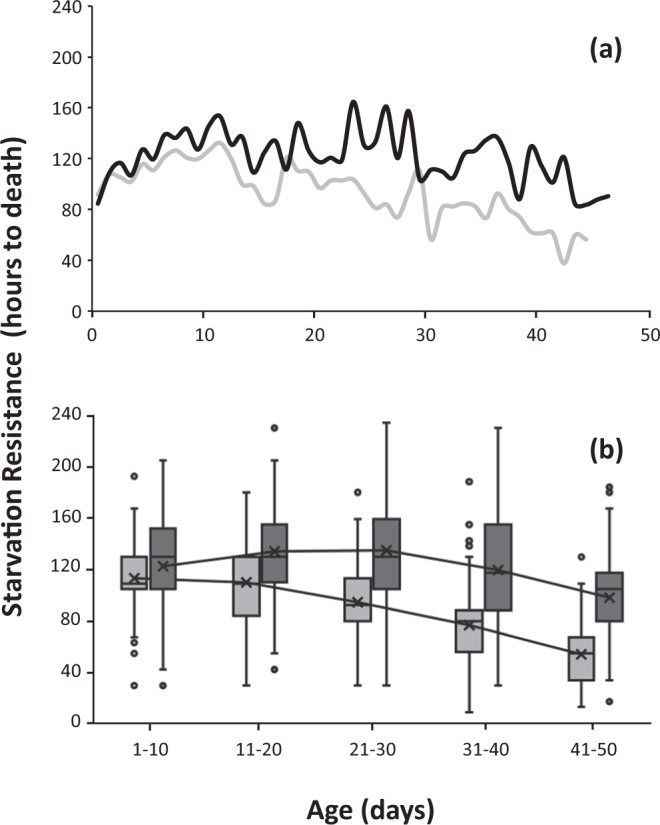
Age-specific curves (**a**) and boxplot distribution (**b**) of SR in citrus reared *Ceratitis capitata* flies and kept on adult diet restriction. Light grey bars indicate males and dark grey indicate females respectively. Lines along the boxplots connect the mean time to death among each age class.

Age-specific SR in citrus reared medflies kept on adult diet restriction followed a different pattern fluctuating across the lifespan (Fig. 2a). Initially, an increase in SR was observed within the first 15 days for both males and females (Fig. 2a). From the mean time to death line in Fig. 2b we observe that SR decreased with male age, while for females SR increased up to the age of 30 days and then slowly dropped in older ages. Overall, age was a significant predictor of SR (Wald X^2^ = 41.54 df = 4, P = 0.0001). However, the distribution of SR in each age class (Fig. 2b) and pairwise comparisons among age classes show that flies up to 20 days old did not differ in their SR, while differences were present in all other age-classes (Supplementary Table 2). Females are more resistant than males to food absence, irrespective of their age (X^2^ = 65.02 df = 1, P = 0.0001).

**Figure 3:**
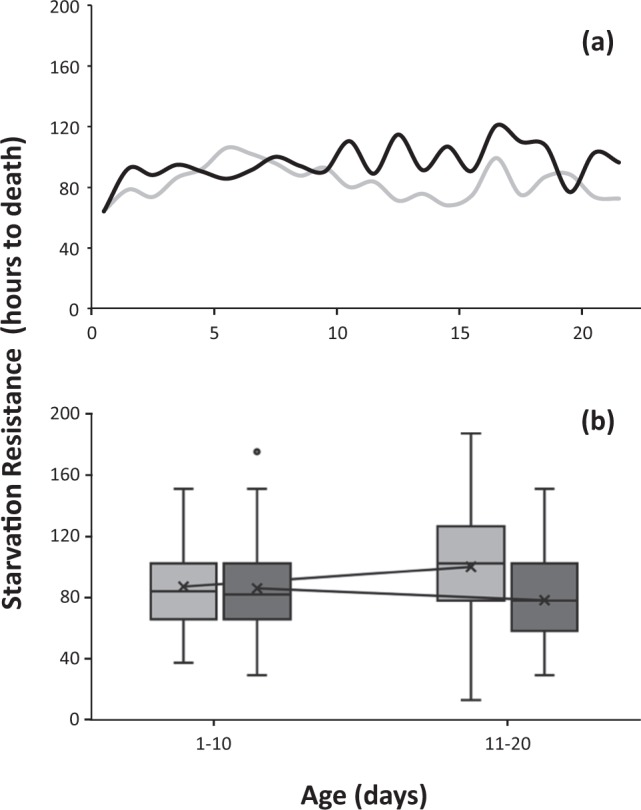
Age-specific curves (**a**) and boxplot distribution (**b**) of SR in *Ceratitis capitata* flies reared in artificial larval diet and kept on adult diet restriction conditions. Light grey bars indicate males and dark grey indicate females respectively. Lines along the boxplots connect the mean time to death among each age class.

In diet restricted adults that were reared on the artificial diet, SR increased within the first 5 days after emergence for both males and females (from 70 to ≈ 100 hours, Fig. 3a). However, from days 5–20, females managed to further increase their resistance, whereas SR in males dropped to the initial level. The mean time to death line in the boxplot (Fig. 3b) shows the decreasing and increasing trend in males and females respectively. Overall, age was a significant predictor (Wald X^2^ = 10.161, df = 2, P = 0.006) and females were more resistant than males (X^2^ = 8.37 df = 1, P = 0.004).

**Figure 4:**
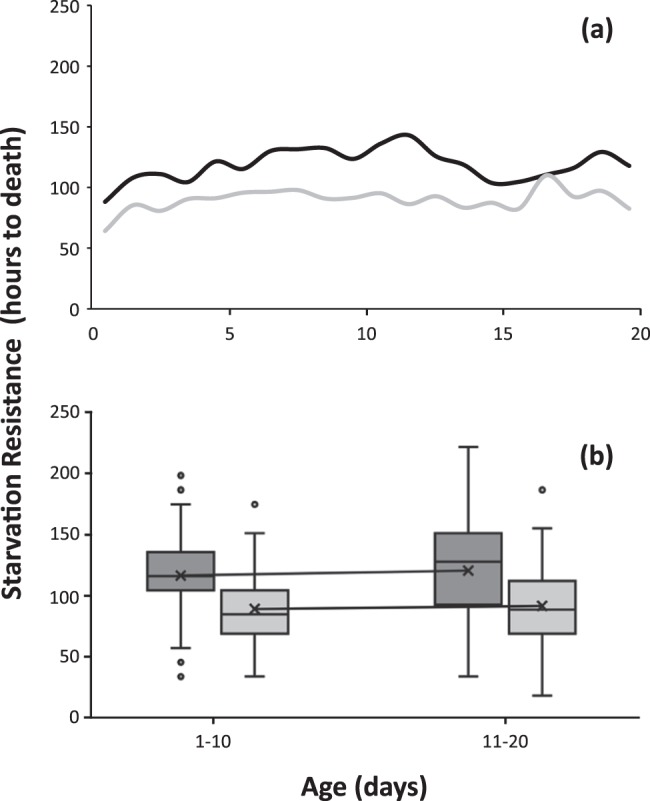
Age-specific curves (**a**) and boxplot distribution (**b**) of SR in citrus fruit (black line) and artificial larval diet (grey line) reared *Ceratitis capitata* flies, kept on adult diet restriction conditions. Lines along the boxplots connect the mean time to death among each age class.

Citrus reared flies resisted starvation more than flies reared on artificial diet and kept in adult diet restriction (Fig. 4a, Wald X^2^ = 61.188, df = 1, P = 0.001). SR increased slightly across the first 20 days of all medflies (irrespective of their larval diet), but significant differences were only observed between the two age-classes in citrus reared medflies (Fig. 4b and Supplementary Table 4). Again, females were more tolerant than males in both developmental substrates (X^2^ = 26.43, df = 1, P < 0.001).

**Figure 5:**
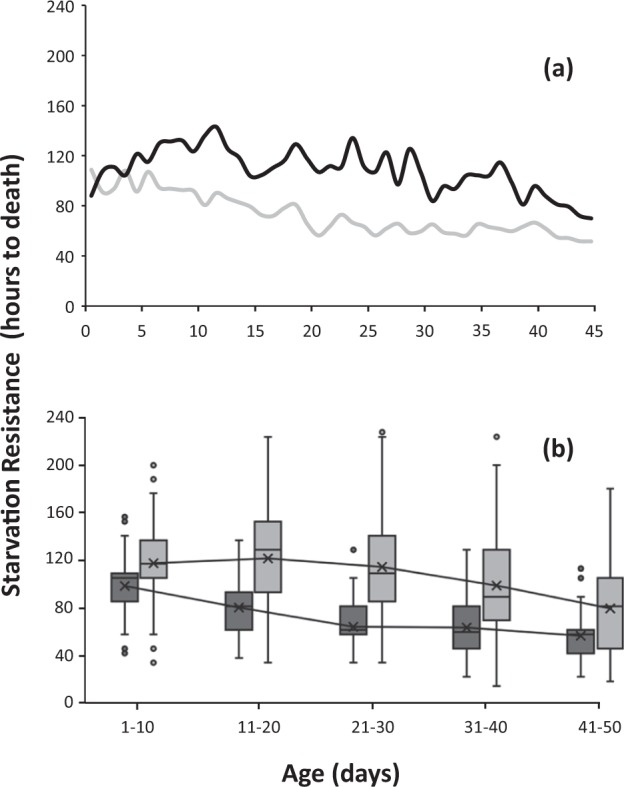
Age-specific curves (**a**) and boxplot distribution (**b**) of SR in citrus reared *Ceratitis capitata* flies and kept on full adult diet (black line) and adult diet restriction conditions (grey line). Lines along the boxplots connect the mean time to death among each age class.

Comparisons between full diet and diet restricted adults reared in citrus revealed that Mediterranean fruit flies kept in adult diet restriction exhibited a greater SR across the whole age spectrum (Fig. 5a, Wald X^2^ = 112.46, df = 1, P = 0.001). SR in adult diet restricted medflies exhibited a fluctuating pattern, while on adult full diet a decreasing trend from the beginning and as flies aged was observed (Fig. 5a). Age was a significant predictor of SR in both adult diets (Wald X^2^ = 96.106, df = 6, P < 0.001) and SR differed among most of the age-classes (Fig. 5b, Supplementary Table 5). Again, females exhibited higher SR than males (Wald X^2^ = 24.91, df = 1, P < 0.001) in both adult diets.

### Significant determinants of starvation resistance

The overall non-parametric proportional hazard model that was performed (using pooled data) showed that all of the factors tested and most of their interactions had significant effects on shaping starvation resistance. In particular, age (X^2^ = 10.31,df = 1, P = 0.0013), adult diet (X^2^ = 558.90, df = 1, P < 0.001), larval diet (X^2^ = 9.44 df = 1, P = 0.0021), gender (X^2^ = 156.25, df = 1, P < 0.001), ageXgender (X^2^ = 11.62, df = 6, P < 0.001) and ageXlarval diet (X^2^ = 10.69, df = 1, P < 0.001) were significant predictors of starvation resistance. Only ageXadult diet interaction was not statistically different (X^2^ = 1.44, df = 1, P = 0.22). To rank these variables and identify the most important predictors of SR, a stepwise regression model optimization procedure was performed^25,26^. The stepwise regression selected the best fitting model by automatically adding or removing individual predictors, a step at a time, based on their statistical significance. This procedure revealed that from the variables tested, age and adult diet were the most significant, followed by gender and the larval diet (Supplementary Tables 6 and 7). The optimized AFT regression model that we used was: *lnS*R = 4.871 + 0.01X1 − 0.294X2 − 0.1X3 − 0.04X4 + 0.0001X4^2^ + 0.221X1 * X3 + 0.001X1 * X4 − 0.114X2 * X3 + 0.01X2 * X4  (Y = lnSR, X1 = adult diet, X2 = larval diet, X3 = gender, X4 = age) and explained the 42.5% of the variation in lnSR.

### Starvation resistance and breaking points

**Figure 6:**
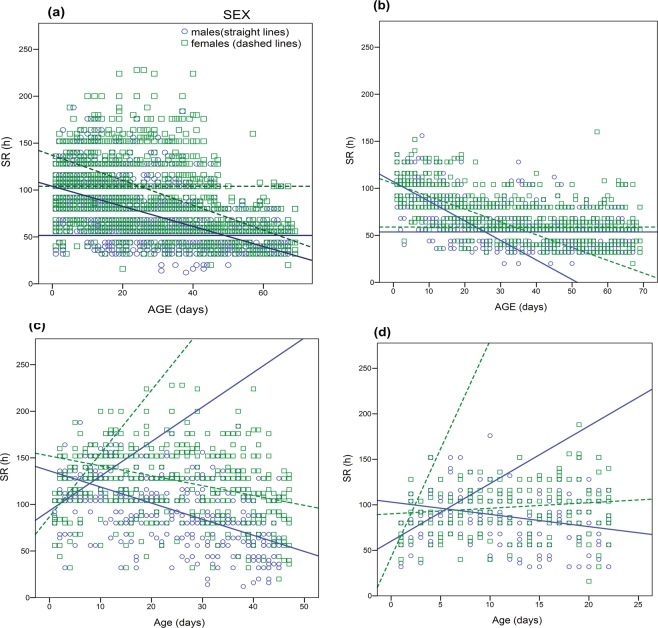
Segmented linear regression with optimum break points (BP) between age and SR for male (blue line) and female (green line) *Ceratitis capitata* flies. (**a**) Shows results of pooled data. (**b,c**) Refer to citrus reared *Ceratitis capitata* flies kept on adult full diet (**b**) and adult diet restriction (**c**) conditions, whereas (**d**) shows results of *Ceratitis capitata* flies reared on artificial larval diet and kept on adult diet restriction.

The segmented regression analysis showed that the breaking point (BP) as well as the slope of SR varies between males and females and among different treatments. Pooled data analysis identified the BP at the age of 25 and 48 days for females and males respectively (Fig. 6a). However, BPs differed when data were analyzed separately (in each treatment). In citrus reared medflies, the BPs were identified at 34 and 25 days for females and males kept in full diet respectively (Fig. 6b), while for diet restricted medflies the BP was estimated at ≈ 8 days for both sexes (Fig. 6c) In diet restricted medflies that were reared in artificial diet, the BP was identified at 2 and 5.6 days for females and males, respectively (Fig. 6d).

## Discussion

Our results demonstrate that (a) SR differentially changes with age in relation to other variables, (b) diet restricted adults are more tolerant to starvation than individuals kept in adult full diet, (c) females are more tolerant than males, (d) all the tested factors (i.e. age, gender, adult diet and larval diet) have significant effects in shaping SR but of a different magnitude, (e) SR assays may provide insights into the onset of functional ageing in organisms. Therefore, our major hypothesis that starvation resistance will decline with age should be accepted.

To our knowledge this is the first and unique study in starvation literature (beyond *Drosophila*) that investigates how SR changes across each day and for the whole spectrum of the adult lifespan. Our experimental approach provided a lifetime thorough measurement of medflies starvation resistance, facilitating the production of age-specific SR figures and thus enabling us to extract valuable information on this trait. However, the design of this study does not consider how flies in the different treatments being compared may behave (i.e. showing variation in spontaneous behavioural activity levels that may be driving some of the differences). For example, males and females of *Bactrocera tryoni* and *Drosophila melanogaster* of the same age exhibit different patterns of spontaneous activity, which has been interpreted to reflect differences in life history and physiological requirements of the sexes^27–29^. There are numerous studies in the literature describing how stress resistance characters are shaped in relation to age and to other factors that could be commented in our discussion (for reviews see^9,30^). However, because it is not our aim to provide a comprehensive review on stress resistance, but to give some original and fresh data on SR, we comment on studies that only and clearly refer to starvation resistance.

### Age- and adult diet-specific effects

The main goal of this study was to explore how SR changes with age and in relation to other ecological factors. Taking into consideration that adult diet was the other significant factor (after age) that shapes SR and to avoid repetitions, age-specific effects are discussed in relation to adult diet in one section.

Starvation resistance, as expected, varied in relation to age. Yet, it did not follow a consistent and universal pattern and varied among treatments. An age-related decline was observed for citrus reared adults kept in full diet early in their adult life and until the age of 40 days, whereas for the rest of their lifespan SR remained relatively stable (Fig. 1a and Supplementary Table 1). This pattern is partly (due to different experimental approaches) in agreement with studies on *Drosophila* that report a general decline tendency in SR within the first 15 days of the adult life in virgin females^31^, while recently, Everman^20^, comparing SR in 1- and 4-weeks old *Drosophila* flies concluded that average SR declines with age in both sexes and that the phenomenon is more pronounced in females.

An overall decline was also reported between young (1–10 days) and older (40–50 days) citrus reared medflies kept on adult diet restriction. From days 1–15 there was an increase in SR, which ceased for males but continued for females until the age of 30 days. From that time point and thereafter, SR decreased significantly for both sexes, especially for males (Fig. 2 and Supplementary Table 2). This fluctuation in SR on adult diet restriction is concordant to studies on *Drosophila* flies that report an increase in SR when feeding in a nutritionally low medium (i.e. without yeast)^19,32,33^. A similar increase was also recorded in early ages, if we consider that the studies on *Drosophila* measure SR for a short period of the adult lifespan, while we monitored SR for the whole adult lifespan and managed to detect the fluctuations in SR that took place in older ages. If we focus on the specific time window of early to mid-ages (from pupa eclosion up to 30–35 days adult age), the similarity is obvious.

In diet restricted medflies that were developed on the artificial diet a reverse pattern in SR was observed, with age positively affecting SR in females and without any effects on males (Fig. 3 and Supplementary Table 3). However, we have to point out that these effects are observed only until the age of 22 days (because of logistics issues) and perhaps what we observe is an artifact of the experiment. If the starvation assays were continued for older ages, a similar pattern with that of diet restricted adults developed in citrus fruits could exist (because of similarities that exist in early ages between the two groups of flies).

Although it was not an objective of this study to document which strategy medflies might employ to overcome SR, we provide some explanations. Ageing is known to regulate metabolism in insects^34,35^. Generally, metabolism is expected to decrease with adult age, due to functional senescence and deterioration of physiological condition^10^. However, some studies report an increase or no change in metabolism and it seems that the ultimate outcome also varies in relation to other parameters (e.g. food, seasonality, body mass). In this study, ageing, along with rich adult diet conditions (i.e. full diet), could activate a higher metabolism in individuals leading to a faster depletion of energy reserves. It is possible that metabolism increases as the number of days that adults feed accumulates and thus explains the decreasing pattern in SR observed in full diet conditions. The stabilization observed above 40 days could be due to a plateau in the elevation of metabolism in medflies that is achieved at that age. Plateaus in stress resistance characters have been demonstrated empirically and theoretically in several studies (discussed in detail by^18,36^). This scenario also comes in accordance with the pattern observed in adult diet restricted flies. Diet restricted flies might have a low metabolism early in their life, due to their poor (caloric) adult diet (and thus high SR), while their metabolism could be elevated as the days of feeding accumulate.

The other possible strategy, which is not mutually exclusive from the above, is that young medflies are more resistant than older ones, because early in life, they possess greater energy reserves (mainly lipids) to direct towards soma maintenance. Medfly adults are capable of synthesizing lipids and their lipid levels have been found to decrease as individuals age^37,38^, and oscillate harmonically with a certain periodicity^39^. These studies showed that body lipids declined in adults experiencing short term starvation, providing evidence that the lipid mechanism is involved in SR in the medfly^37,38^. Furthermore, Weldon exploring SR and other stress resistance characters among *C. capitata* populations found a link between high SR and high body lipids, enhancing the general conception that lipids reservoirs can be a strong source of variation for SR in the medfly^28,40^.

Differences in age-specific SR between full diet and diet restricted adults could be due to the differential ability of medflies to build such nutritional reservoirs^37^. In *Drosophila melanogaster,* adults fed on protein-poor diet accumulate larger amounts of body lipids than those fed on protein-rich diet^41^. However, the positive effects of poor adult diet are reversed under prolonged feeding periods^42^.

Our results follow a similar pattern; high SR is observed in adult diet restricted Mediterranean fruit flies early in their life, which is followed by a decline with ageing (Fig. 5 and Supplementary Table 5). However, contrary to *Drosophila*, adult diet restricted medflies exhibited higher SR than those kept in adult full diet, even when poor feeding was continued for the whole adult lifespan. Such discrepancies, could be due to the fact that in our experiment, adults did not have access to a yeast-hydrolysate (protein) source, while Burger^42^ manipulated the amount of yeast provided to *Drosophila* flies. Even small amounts of yeast could evoke the expression of different genes and thus shape the expression of several functional traits. The decline observed in adult diet restricted flies at old ages could be either due to elevation of their metabolism after prolonged “compensational” feeding or physiological deterioration due to ageing (as mentioned above). Medflies may also employ both of these strategies.

### Larval diet specific effects

Adult diet restricted medflies developed in citrus fruits exhibited higher SR than those reared on the artificial diet (Fig. 4 and Supplementary Table 4). Although our data enable comparison between the two different treatments only within the first 20 days of the adult life, yet our results provide a clear picture and represent a significant time-period (at least ecologically) of the Mediterranean fruit fly’s adult life.

The higher SR of medflies reared on citrus could be due to ontogenetic carry over effects from the larval stage. In *Drosophila* ontogenetic effects have been proposed as a possible explanation for the patterns observed in several stress resistance traits across early adult life (see^11^ for an extended discussion). Nutrients acquired during larval stages can be stored in the fat cells and may shape adult stress resistance, as shown in insect species^43,44^. Likewise, emerging medfly adults developed in citrus fruits may carry greater lipid reserves already at eclosion because of the nutrient composition of citrus fruits. Larval diet composition (protein: carbohydrate ratio) affects weight and lipid reserves in pupating *C. capitata* larvae^45,46^, while citrus reared flies may have access to exclusive nutrients found in the fruit (e.g. amino acids as a product of symbiotic bacteria and larval flies, as discussed in^46^); this could potentially favor higher lipid levels and thus higher SR.

Another possible explanation is that higher SR in citrus reared medflies could arise from the longer developmental period that these individuals experience. *C. capitata* larvae developing in natural hosts require a longer period to pupate than those developing on the artificial diet^47^. In our study, average larvae developmental time (i.e. egg to pupa) was ≈ 13 days for flies reared in bitter oranges and ≈8 days for medflies reared in artificial larval diet. Generally, prolonging the period of feeding is a simple way for the larvae to accumulate additional nutritional reserves that could be utilized to enhance SR under unpredictable food conditions^3^.

### Gender-specific effects

Females had higher SR than males in all treatments and across the entire adult lifespan. This is in line with the majority of other studies exploring SR in insects. Females could endure more because of greater energy reserves stored in their larger body size, as has been shown for other insect species^13,14,48,49^. Although, Nestel and co-workers showed that in the Mediterranean fruit fly lipid levels between males and females were similar^39^, yet the different experimental approach and the strain of flies they used, does not allow us to make safe comparisons. Here, individually caged wild flies were used, while Nestel sampled flies (for lipid analysis) from large mass reared cohort cages, containing both sexes^39^. In such cages, flies would most probably interact with each other, and their physical activity, could have driven their results. The genetic background and evolutionary history of the different strains used in each study could also result in such discrepancies. Furthermore, considering that body size and lipids correlate positively in laboratory reared *C. capitata*^28^, and that females are bigger than males in this species, the sex-specific differences in SR are easily explained.

### Variable ranking

The analysis revealed that age and adult diet were the most significant determinants of SR, followed by gender and the larval diet. This is partially in accordance with our hypothesis, with the exception of larval diet, which proved not to be such a strong determinant of SR as we expected. Although larval diet (during development) evokes multifaceted effects in adult insects and in *C. capitata*, affecting reproduction, ageing and tolerance to stress^43,50–52^, yet in our case it proved to be a weak predictor of SR. In our experiment we have chosen to rear larvae in optimum conditions in relation to larval diet availability and crowding conditions, so as to explore differences between the developmental environments (natural VS artificial larval diet), rather than investigate the detailed nutritional profile of each larval diet and the consequences to SR. These optimal conditions within the artificial larval diet may have weakened the overall significance of larval diet. Furthermore, in our study the effects of larval diet were explored only up to the age of 22 days and in adults kept in diet restriction. The inclusion of data of older age individuals reared on artificial diet and fed on full diet might have changed the overall significance of larval diet.

### Breaking points and plateaus in starvation resistance

Our primary interest in the starvation data was to provide evidence whether SR can be used as a biomarker of ageing. The results reveal interesting information about SR across the medfly’s lifespan. However, due to the high variation and because of the inconsistent patterns observed, we cannot confidently support the notion that SR can predict ageing.

Our intention to use breaking points was not to give the best fitting model but rather to provide an estimate of the breaking point (i.e. time point in lifespan at which SR begins to stabilize or change abruptly and potentially corresponds to significant physiological processes related to ageing and thus depict the onset of senescence).

Pooled analysis identified the breaking points (BPs) at the age of 28 and 48 days for females and males respectively (Fig. 6). Although pooled analysis results in a larger sample, which is better for estimating SR patterns, yet we decided to also estimate the BP for each treatment separately, because SR proved to be a context dependent trait (see previous sections). The breaking points in medflies kept in full adult diet were estimated at 25 and 34 days for males and females respectively, while in adult diet restricted the BP was estimated at 8 days for both sexes. We do not have a solid explanation for the difference in the BPs between full adult diet and diet restricted flies. We believe that such differences possibly arise from early life nutrition, which is known to evoke long lasting effects to physiology in animals. Our BPs are very close to similar parameters used in other studies. In *Drosophila* the parameter breakday, which describes the age at which functional characters stabilize, has been reported at around 35 days^18^.

SR plateaus have been identified in our study. It is the first time that plateaus in fitness traits, other than mortality and fecundity, have been identified in the medfly. Plateaus were present in both sexes and both diets, differed on the exact period of initiation and were depicted across a significant period of the medfly’s lifespan. Plateaus in SR have been shown in *Drosophila* fruit flies and have also been demonstrated for other fitness traits^53,54^. Although it is beyond the scope of this article, SR plateaus depicted constitute supporting evidence for the lifelong heterogeneity theory (i.e. variation in trait robustness among individuals results in population filtering across lifespan, leading to the emergence of late-life mortality plateaus) and should be tested in future studies. However, it must be pointed out that our results could also be explained by alternative theories, such as the evolutionary Hamiltonian theory and late-life physiology detailed in^55^.

### Implications of the study

Undoubtedly, SR can define the ability of an invasive generalist with complex life history traits to successfully colonize diverse habitats. A better understanding of SR may provide significant information on the population dynamics of this species, components that are especially important when building models to predict the expansion of this species, but also the population –level outcomes of control measures.

Furthermore, among the practical uses of any findings are applications to control methods used against insects of medical and agricultural significance via mass reared, sterilized male insects released in the field to compete against wild males for wild females (SIT). The efficacy of this method depends greatly on fitness traits (behavioural and physiological) of mass reared sterilized males compared with those of wild males. Thoroughly investigating adult stress resistance in insects may ultimately allow researchers to modify rearing (early life) conditions of sterile insects to improve desirable physiological traits of sterile mass reared adult insects (e.g. starvation and desiccation resistance). In particular, our results show that adopting an adult diet containing only sugar, in the sterile insect technique (which is also more cost-effective compared to yeast-hydrolysate diets) might strongly enhance SR and potentially other fitness traits in medfly, ultimately affecting the efficacy of the method. However, we have to point out that under SIT conditions some other factors (e.g. irradiation, crowding, courtship rituals, etc.) with strong impact take place that can heavily moderate the effects that we observed in our study. Future studies may build on these findings and attempt to elucidate the mechanistic basis of SR in the medfly, ultimately providing fundamental and applied benefits and thus benefit human welfare.

## Materials and Methods

### Flies used and treatments

The experiments, as well as the fly rearing were performed, under constant laboratory conditions at 25 ± 2 °C, 65 ± 5% RH, and a photoperiod of L14:D10. Adults originating from larvae developed in bitter oranges, and from larvae reared on an artificial larval diet were used (synthesis of the artificial larval diet is given in the Supplementary Table 8 as first described by^56^).

Rearing was performed in relaxed conditions to minimize any confounding effects potentially arising from crowding. Specifically, care was taken to control the number of: (a) eggs placed in specific amount of larval food (i.e. 30 eggs in 3 ml of larval diet, Diamantidis & Papadopoulos personal communication), (b) oviposition stings (not clutch sizes) to 2 per bitter orange. Clutch sizes (i.e. number of eggs per oviposition sting) in *Ceratitis capitata* usually range between 1–10 eggs^57^ and one bitter orange can sufficiently support the development of at least 20 eggs^58^. Adults initially originating from pupae that were derived from infested fruits, developed in bitter oranges for one generation in the laboratory to exclude any maternal effects. We used recently collected flies because resistance to environmental stress has been found to weaken or even become entirely lost under laboratory adaptation in other insects^59,60^. Laboratory domestication has been shown to affect key biological traits in the medfly and thus potentially starvation resistance^61^.

Emerging adults (of both sexes) were caged individually (male or female) to exclude any crowding and social interaction effects. All flies were unmated to minimize any confounding effects; mating has been found to boost egg production in protein-fed females with a subsequent cost in survival^62^ and this would potentially result in other trade-offs among fitness traits, including starvation. For citrus reared larvae, half of the emerging adults were fed on a mixture of hydrolyzed yeast and sugar (full adult diet conditions), whereas the other half were fed only on sugar (adult diet restricted). For artificial diet reared larvae adults fed only on sugar were tested (due to logistics issues). Water was provided in all cohorts.

### Starvation assays

From the first day and for each day of their adult life (including the day of emergence) 10 males and 10 females were randomly selected from each treatment, transferred to new individual plastic cages containing only water (to eliminate any possible feeding on faeces/excreta deposited on the plastic surface of cages from the previous days) and tested for their resistance to food absence. SR was measured in time (hours) required for each individual to die. Mortality was scored every 4 hours until all the flies were dead.

### Statistical analysis

We first performed pairwise comparisons using Kaplan-Meier log rank tests, to determine where the differences lie among the age groups, between males and females and different cohorts. We also followed a mix-model approach, using semi-parametric and parametric survivorship models to partition variation in SR among the tested factors. Particularly, a proportional Hazard (PH) model and an Accelerated Failure time (AFT) type of regression model as complement, were used to explore for main and interaction effects among medfly’s age, adult diet, larval diet and sex in the different cohorts, but also overall. Additionally, to rank the impact of the tested variables in shaping SR a stepwise regression model optimization procedure was performed based on the AFT model, having the natural logarithm of SR as the dependant variable and with the residual errors following the Weibull distribution^63^. Finally, to investigate if SR may act as a biomarker of ageing (i.e. to detect the ‘’breakpoint” age, referred also as BP), we performed a segmented regression between SR and age. In particular, the best fitting linear functions were found by maximising the statistical coefficient of explanation for different BPs (including the correlation coefficient found when omitting BPs)^64^. Ideally, SR should assay the biological process of ageing, and should be reproducibly measurable during a short interval compared to the life of the organism. Data of flies, emergence day 0, (without any feeding) are not included in the analysis, so as not to confound readers but will be given in the Raw data material uploaded in open repository.

## Supplementary information

Supplementary material_SciRep_Revised

## Data Availability

Data will be deposited in a digital repository.
