# Supplementary material for: Age, sex, adult and larval diet shape starvation resistance in the Mediterranean fruit fly: an ecological and gerontological perspective
Source: Sci Rep. 2019 Jul 24;9:10704. doi: 10.1038/s41598-019-47010-0 (PMC6656776; doi:10.1038/s41598-019-47010-0)
Supplement: Supplementary file 1 — Supplementary material_SciRep_Revised [file 41598_2019_47010_MOESM1_ESM.docx]

**Submitted to:**

Scientific Reports

**Age, sex, adult and larval diet shape starvation resistance in the Mediterranean fruit fly: an ecological and gerontological perspective**

*Christos D. Gerofotis^1,^ Nikos A. Kouloussis^1*^, Christiana Koukougiannidou^1^, Nikos T. Papadopoulos^2^, Petros Damos^1^, Dimitris S. Koveos^1^ and James R. Carey^3,4^*

^1^ Laboratory of Applied Zoology and Parasitology, School of Agriculture, Aristotle University of Thessaloniki, 54124 Thessaloniki, Greece

^2^ Laboratory of Entomology and Agricultural Zoology Department of Agriculture Crop Production and Rural Environment, University of Thessaly, Phytokou St. 38446 N. Ionia Volos, Greece

^3^ Department of Entomology, University of California, Davis, CA 95616, United States

^4^ Center for the Economics and Demography of Aging, University of California, Berkeley, CA 94720, United States

***^*^Correspondence to:***

*Nikos A. Kouloussis*

[*nikoul@agro.auth.gr*](mailto:nikoul@agro.auth.gr)

Laboratory of Applied Zoology and Parasitology, School of Agriculture, Aristotle University of Thessaloniki, 54124 Thessaloniki, Greece

**Supplementary information**

Supplementary Table 1. Average hours to death and pairwise comparisons (Log-rank, Kaplan-Meier test) for flies reared in citrus fruits and kept in full diet conditions as adults.

| Mean hours to death | | | | | | | |
| --- | --- | --- | --- | --- | --- | --- | --- |
| ***Age class*** | | | | | | | |
|  | ***1-10*** | ***11-20*** | ***21-30*** | ***31-40*** | ***41-50*** | ***51-60*** | ***61-70*** |
| Males | 96.20±1.92A(a) | 73.24±1.92B(a) | 56.96±1.92CD(a) | 57.92±1.90C (a) | 51.52±1.92DE(a) | 53±1.92DE(a) | 50.17±2.29E(a) |
| Females | 98.52±1.92A(a) | 84.56±1.92B(b) | 68.12±1.92C(b) | 65.72±1.92CD(a) | 59.76±1.92E(b) | 56.8±1.92E(a) | 55.07±1.92E(a) |

Different capital letters show significant differences among age classes within the same sex, whereas small letters in brackets indicate significant differences between the two sexes for the same age-class (P<0.005).

Supplementary Table 2. Average hours to death and pairwise comparisons (Log-rank, Kaplan-Meier test) for flies reared in citrus fruits and kept in diet restriction conditions as adults.

|  | Mean hours to death | | | | |  |
| --- | --- | --- | --- | --- | --- | --- |
|  | ***Age class*** | | | | |  |
|  | ***1-10*** | ***11-20*** | ***21-30*** | ***31-40*** | ***41-50*** |  |
|  | Males | 112.04 ± 2.65A(a) | 108.92 ± 3.26A(a) | 94.08 ± 2.96B(a) | 76.98 ± 2.94C(a) | 55.20 ± 3.62D(a) |
|  | Females | 120.94 ± 3.26A(b) | 132.16 ± 3.54B(b) | 132.80 ± 3.71B(b) | 118.20 ± 3.86A(b) | 97.48 ± 3.97C(b) |

Different capital letters show significant differences among age classes within the same sex, whereas small letters in brackets indicate significant differences between the two sexes for the same age-class (P<0.005).

Supplementary Table 3. Average hours to death and pairwise comparisons (Log-rank, Kaplan-Meier test) for flies reared in artificial diet and kept in diet restriction conditions as adults

| Mean hours to death | | | |
| --- | --- | --- | --- |
| ***Age class in days*** | | | |
|  | ***1-10*** | ***11-20*** | ***21-30*** |
| Males | 87.88 ± 2.66A(a) | 80.18 ± 2.65A(a) | 73.0 ± 5.72B(a) |
| Females | 89.08 ± 2.45A(a) | 101.82 ± 2.98B(b) | 99.6 ± 7.27B(b) |

Different capital letters show significant differences among age classes and within the same sex whereas different small letters in brackets indicate significant differences between the two sexes for the same age-class (P<0.005).

Supplementary Table 4. Average hours to death and pairwise comparisons (Log-rank, Kaplan-Meier test) for medflies developed in citrus fruits and between flies kept in full diet and diet restriction conditions.

| Mean hours to death | | | | | | | |
| --- | --- | --- | --- | --- | --- | --- | --- |
| ***Age class*** | | | | | | | |
|  | ***1-10*** | ***11-20*** | ***21-30*** | ***31-40*** | ***41-50*** | ***51-60*** | ***61-70*** |
| FD | 97.36 ±1.50A(a) | 78.90 ± 1.66B(a) | 62.54 ± 1.21C(a) | 61.82 ± 1.47C(a) | 55.64 ± 1.29D(a) | 54.9 ± 1.27D | 52.92 ± 1.34D |
| DR | 116.49±2.12A(b) | 120.54 ± 2.54B(b) | 113.44 ± 2.74AB(b) | 97.59 ±2.82C(b) | 79.86 ± 3.35D(b) |  |  |

Different capital letters show significant differences (P<0.005) among age classes and within the same adult diet, whereas different small letters in brackets indicate significant differences (P<0.005) between the two different adult diets for the same age-class

Supplementary Table 5. Average hours to death and pairwise comparisons (Log-rank, Kaplan-Meier test) for dietary restricted adult medflies and between flies developed in citrus fruits and in artificial diet conditions.

| Mean hours to death | | | |
| --- | --- | --- | --- |
| ***Age class in days*** | | | |
|  | ***1-10*** | ***11-20*** | ***21-30*** |
| Citrus | 116.49 ± 2.12A(a) | 120.54 ± 2.54B(a) | 73.0 ± 5.72B(a) |
| Artificial | 88.48 ± 1.80Α(b) | 91 ± 2.13Α(b) | 86.3 ± 5.04Α(b) |

Different capital letters show significant differences (P<0.005) among age classes and within the same developmental environment (larval diet), whereas different small letters in brackets indicate significant differences (P<0.005) between the two different larval diets for the same age-class.

Supplementary Table 6 Effect likelihood ratio test of the non-parametric proportional hazard model (response variable: starvation resistance, total observation used 2720, overall model performance test: ChiSquare: 1310.591, df=9, Prob>Chisq:<0.0001)

| **Source** | **Nparm** | **DF** | **L-R ChiSquare** | **Prob>ChiSq** |
| --- | --- | --- | --- | --- |
| Adult diet | 1 | 1 | 558.903991 | <.0001* |
| Larval Diet | 1 | 1 | 9.44394988 | 0.0021* |
| Gender | 1 | 1 | 156.250865 | <.0001* |
| Ageclass | 1 | 1 | 10.3155333 | 0.0013* |
| Ageclass*Gender | 1 | 1 | 11.628687 | 0.0006* |
| Ageclass*Adult Diet | 1 | 1 | 1.44564559 | 0.2292 |
| Ageclass*Larval Diet | 1 | 1 | 10.9628312 | 0.0009* |

* Significant at a=0.05 level

Supplementary Table 7**.** Effect likelihood ratio test of the accelerated failure time (AFT) model (response variable: starvation resistance, total observation used 2720, overall model performance test: ChiSquare: 1645.028, df=9, Prob>Chisq: <0.0001, distribution: Weibull, -2*LogLikelihood: 25348.46)

| **Source** | **Nparm** | **DF** | **L-R ChiSquare** | **Prob>ChiSq** |
| --- | --- | --- | --- | --- |
| Adult Diet | 1 | 1 | 724.098012 | <.0001* |
| Larval diet | 1 | 1 | 11.9870788 | 0.0005* |
| Gender | 1 | 1 | 190.776751 | <.0001* |
| Ageclass | 1 | 1 | 12.4111863 | 0.0004* |
| Ageclass*Gender | 1 | 1 | 14.012462 | 0.0002* |
| Ageclass* Adult Diet | 1 | 1 | 2.84603427 | 0.0916 |
| Ageclass* Larval diet | 1 | 1 | 12.7485047 | 0.0004* |

* Significant at a=0.05 level

Supplementary Table 8. Composition of the artificial larval diet that was used in our experiments^56^.

| 100 gr solid sugar |
| --- |
| 100 gr |
| 50 gr soya bean |
| 8 gr citric acid |
| 8 gr ascorbic acid |
| 2 gr mixture of salts |
| 1.5 gr sodium propionate |
| 500 ml of water |
